# Co-aggregation of annexin A11 and TDP-43 in FTLD/MND with primary lateral sclerosis phenotype

**DOI:** 10.1186/s40478-025-02210-w

**Published:** 2026-01-04

**Authors:** Airi Tarutani, Takashi Nonaka, Reiko Ohtani, Kazunori Imai, Yasuhiro Ito, Hiroshi Tsuji, Akihide Mochizuki, Akira Tamaoka, Tetsuaki Arai, Andrew C. Robinson, David M. A. Mann, Takayuki Kosaka, Hitoshi Takahashi, Akiyoshi Kakita, Mari Yoshida, Masato Hasegawa

**Affiliations:** 1 Department of Clinical Medical Sciences, Tokyo Metropolitan Institute of Medical Science, Tokyo, 156–8506 Japan; 2 Department of Neurology, National Center for Geriatrics and Gerontology, Aichi, 474–8511 Japan; 3 Department of Neurology, Toyota Memorial Hospital, Aichi, 472–8513 Japan; 4 Department of Neurology, Graduate School of Medicine, Osaka Metropolitan University, Osaka, 545–8585 Japan; 5 Ibaraki Health Service Association, Ibaraki, 310–0852 Japan; 6 Department of Neurology, Tsukuba Memorial Hospital, Ibaraki, 300–2622 Japan; 7 Department of Psychiatry, Institute of Medicine, University of Tsukuba, Ibaraki, 305–8575 Japan; 8 Faculty of Biology, Medicine and Health, School of Biological Sciences, Division of Neuroscience, The University of Manchester, Manchester, M13 9PL UK; 9 Department of Pathology, Brain Research Institute, Niigata University, Niigata, 951–8585 Japan; 10 Department of Neurology, National Hospital Organization Kumamoto Medical Center, Kumamoto, 860-0008 Japan; 11 Department of Laboratory Medicine, Niigata Neurosurgical Hospital, Niigata, 950–1101 Japan; 12 Department of Neuropathology, Institute for Medical Science of Aging, Aichi Medical University, Aichi, 480–1195 Japan

**Keywords:** Primary lateral sclerosis, Amyotrophic lateral sclerosis, TDP-43, Annexin A11, FTLD-TDP

## Abstract

TDP-43 proteinopathies, such as frontotemporal degeneration (FTLD) and amyotrophic lateral sclerosis (ALS), are classified into five neuropathological subtypes, Types A to E, according to the morphology of TDP-43 inclusions. Recent cryo-electron microscopy analysis of FTLD-TDP cases demonstrated that TDP-43 filaments composing the inclusions are structurally different depending on the subtype, and remarkably, co-assembled heteromeric filaments of TDP-43 and annexin A11 (ANXA11) were identified in Type C. Therefore, the involvement of ANXA11 in TDP-43 proteinopathy should be further examined. Here, we pathologically and biochemically analyzed four cases of primary lateral sclerosis-phenotype FTLD/motor neuron disease (MND) with TDP-43 pathology (PLS-TDP), and found that ANXA11 co-localizes with FTLD-TDP Type A pathology in PLS-TDP. Immunoblot analysis of the PLS-TDP cases revealed that the banding patterns of C-terminal and chymotrypsin-resistant fragments of TDP-43 are distinct from those of FTLD-TDP Types A, B and C. In addition, the N-terminal fragments of ANXA11 appear to be different from those of FTLD-TDP Type C. Filaments extracted from PLS-TDP cases were TDP-43- and ANXA11-immunopositive, suggesting the presence of TDP-ANXA11 heteromeric filaments. These results suggest that co-aggregation of ANXA11 and TDP-43 may serve as a neuropathological and biochemical indicator distinguishing PLS from ALS in FTLD/MND.

**Supplementary Information:**

The online version contains supplementary material available at 10.1186/s40478-025-02210-w.

## Introduction

Primary lateral sclerosis (PLS) is a rare motor neuron disease (MND) and is clinically characterized by predominant degeneration of upper motor neurons (UMN) with sparing of lower motor neurons (LMN). The progression of PLS is generally slower than that of amyotrophic lateral sclerosis (ALS), which is a common type of MND in which both UMN and LMN are affected [1–3]. The occurrence of TDP-43 pathology in PLS is infrequent compared with sporadic ALS, in which approximately 97% of cases exhibit TDP-43 accumulation [4–6]. Although ALS frequently overlaps with frontotemporal dementia (FTD), PLS shows this overlap only rarely (~ 3% of cases), which may reflect its relatively limited TDP-43 involvement and distinct clinical profile [4]. PLS is considered to represent part of a continuous spectrum with ALS [7], but criteria to distinguish PLS from ALS have not yet been well established. Therefore, it remains controversial whether PLS constitutes a subtype of ALS or a distinct disease entity.

Frontotemporal lobar degeneration with TDP-43 pathology (FTLD-TDP) is classified into five neuropathological subtypes, according to the morphology of filamentous TDP-43 inclusions [8]. Type A shows neuronal cytoplasmic inclusions (NCIs), short dystrophic neurites (DNs) and occasional neuronal intranuclear inclusions (NIIs). Type B is defined by numerous NCIs with fewer DNs and no NIIs. Type C is characterized by long DNs with fewer NCIs, and Type D displays numerous NIIs and DNs with fewer NCIs. Another subtype, Type E, which is characterized by granulofilamentous neuronal inclusions, has also been proposed [9]. Furthermore, TDP-43 filaments composing the inclusions are biochemically and structurally different depending on the subtype [10–15]. In previous case reports of PLS-phenotype FTLD/MND with TDP-43 pathology (PLS-TDP), the presence of phosphorylated TDP-43 (pTDP-43)-positive NCIs and DNs is often described as Type A, while ALS-TDP shows NCI-predominant Type B pathology [1, 5, 16, 17].

Recently, Robinson et al. performed genetic analysis and immunohistochemistry of numerous autopsied brains and found that annexin A11 (ANXA11) co-aggregates with TDP-43 in Type C cases [18]. ANXA11 inclusions were also very occasionally observed in sporadic and inherited FTLD-TDP Types A and B, ALS and limbic-predominant age-related TDP-43 encephalopathy neuropathologic changes (LATE-NC) cases [18]. Furthermore, a cryo-electron microscopy (EM) analysis by Arseni et al. revealed that TDP-43 and ANXA11 co-assemble in Type C brains [12]. Based on these findings, we immunohistochemically examined TDP-43 proteinopathy cases using pTDP-43 and ANXA11 antibodies and found that Type A pathology in PLS-TDP cases was ANXA11-positive. Further biochemical analysis and immuno-EM demonstrated that insoluble TDP-43 and ANXA11 co-accumulated, appearing to form heteromeric filaments. Our results suggest that co-aggregation of TDP-43 with ANXA11 is a defining characteristic of PLS, which is distinct from ALS, in FTLD/MND with TDP-43 pathology.

## Materials and methods

### Clinical history and neuropathology of PLS-TDP cases

The clinicopathological information for the four PLS-TDP cases used in this study is summarized in Table 1. The clinicopathological characteristics of PLS-TDP cases 1, 3, and 4 have previously been described in detail (case 1 in [19], cases 3 and 4 in [16]). Clinicopathological findings of PLS-TDP case 2 are in preparation for submission as a case report. Other FTLD-TDP cases used in this study are summarized in Supplementary Table 1.

**Table 1.**
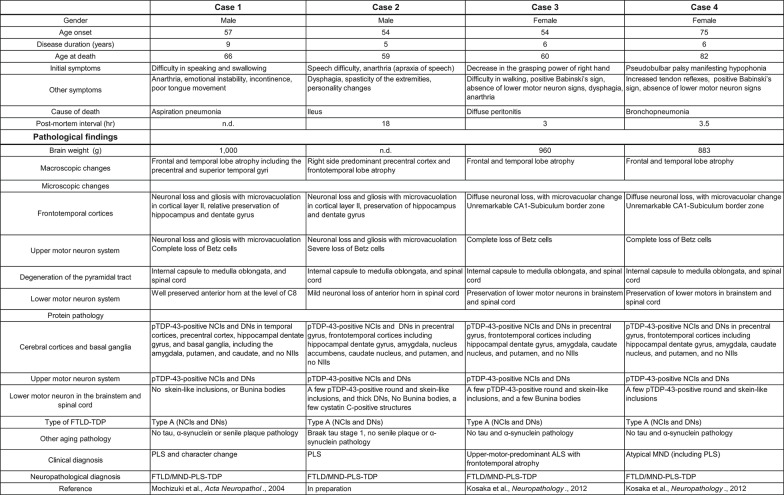
Clinicopathological information of PLS-TDP cases used in this study

pTDP-43, phosphorylated TDP-43; NCI, neuronal cytoplasmic inclusion; DN, dystrophic neurite; NII, neuronal intranuclear inclusion; FTLD, frontotemporal lobar degeneration; PLS, primary lateral sclerosis; ALS, amyotrophic lateral sclerosis; MND, motor neuron disease; n.d., no data

### Immunohistochemistry

Brain tissues were fixed in 10% neutral buffered formalin. Fixed tissues were embedded in paraffin and cut into 8 mm sections. After deparaffinization, the sections from the frontal cortex were autoclaved at 120 °C for 20 min, activated with 100% formic acid for 10 min, treated with 0.5% H_2_O_2_ in PBS for 30 min, and then blocked with 10% calf serum in PBS. Primary antibodies against pTDP-43 (pS409/pS410, CAC-TIP-PTD-M01A, Cosmobio; 1:1,000) and ANXA11 (1–180, 10479-2-AP, Proteintech; 1:1,000) in blocking buffer were used. After treatment with primary antibodies overnight, sections were incubated with biotinylated secondary antibodies (Vector; 1:500) for 2 h at room temperature. Labelling was detected by the avidin-biotin-peroxidase complex (ABC) method using a Vectastain ABC kit (Vector, Burlingame, CA, USA) with 3,3’-diaminobenzidine (DAB) and counterstained with hematoxylin as described [12, 13]. For fluorescence double labelling, mouse monoclonal anti-pTDP-43 (TIP-PTD-M01A, Cosmobio; 1:1,000) and anti-ANXA11 (1:1,000) antibodies and fluorescent-dye-conjugated secondary antibodies (anti-mouse IgG-conjugated Alexa-488 and anti-rabbit IgG-conjugated Alexa-568, Invitrogen; 1:500) were used as described [12]. The sections were analyzed using a LSM 980 confocal laser microscope (Carl Zeiss).

### Genetic analysis

Genomic DNA was extracted from frozen brain tissue. Whole-exome sequencing (WES) was performed using the SureSelectTX Human All-Exon library (v6; Agilent). A HiSeq 4000 (Illumina) was used for sequencing with 75-bp paired-end reads. WES data were analyzed for variants in ALS/FTD-associated genes, including *ANXA11*, *SOD1*, *SETX*, *FUS*, *PGRN*, *VAPB*, *ANG*, *TARDBP*, *FIG4*,* OPTN*, *VCP*, *UBQLN2*, *SQSTM1*, *PFN1*. No pathogenic variants were identified in any gene.

### Filament extraction

The sarkosyl-insoluble fraction was extracted from the frontal cortex of PLS-TDP cases and the temporal cortex of FTLD-TDP Type A, B and C cases, as described [12]. Briefly, 0.5 g of frozen tissue was homogenized with a Polytron in 40 vol (w/v) of extraction buffer consisting of 10% sucrose, 10 mM Tris-HCl, pH 7.4, 0.8 M NaCl and 1 mM EGTA. Homogenates were brought to 2% sarkosyl and incubated for 30 min at 37℃. Following centrifugation at 27,000 g for 10 min at room temperature, the supernatant was spun at 257,000 g for 30 min at room temperature. The pellet was resuspended in extraction buffer containing 1% sarkosyl and centrifuged at 166,000 g for 20 min at room temperature. The resulting pellet was resuspended in 30 mM Tris-HCl (pH 7.4) and used for subsequent analysis.

### Immunoblotting

Immunoblotting was performed as described [13]. Insoluble fractions extracted from 0.02 g of brain tissue were loaded in each lane and separated on 12 or 15% polyacrylamide gels. Proteins were then transferred to a polyvinylidene difluoride (PVDF) membrane and incubated with primary antibodies to pTDP-43 (1:1000) and ANXA11 (ab236599, Abcam; 1:1000). After washing, the membranes were incubated with biotinylated anti-rabbit secondary antibody (Vector; 1:500) for 2 h at room temperature, and the signals were detected using a Vectastain ABC kit with NiCl_2_-enhanced DAB.

### Trypsin and chymotrypsin treatment of the sarkosyl-insoluble fraction

The sarkosyl-insoluble fractions from PLS-TDP and FTLD-TDP cases were sonicated in 30 mM Tris-HCl (pH 7.5), and trypsin or chymotrypsin was added at a final concentration of 10 µg/ml as described [15]. After incubation for 30 min at 37 ℃, the digestion was stopped by adding 2% SDS and boiling, then the digested samples were electrophoresed, and immunoblotted with anti-pTDP-43 antibody.

### Immuno-EM

Immuno-EM was performed as described [20]. Briefly, the sarkosyl-insoluble fractions extracted from 0.03 g of brain tissue of PLS-TDP cases were dropped onto carbon-coated 300-mesh copper grids (Nissin EM) and dried. The grids were immunostained with anti-pTDP-43 antibody or anti-ANXA11 antibody or a mixture of anti-pTDP-43 and monoclonal anti-ANXA11 (1–180, 68089-1-Ig, Proteintech) antibodies for double labelling (1:50), and secondary antibodies conjugated to 5 nm and 10 nm gold particles (Cytodiagnostics, 1:100). The immunostained grids were negatively stained with a drop of 2% phosphotungstic acid and dried. Electron micrograph images were recorded with a JEOL JEM-1400.

## Results

### ANXA11-positive FTLD-TDP Type A pathology in PLS-TDP cases

**Fig. 1:**
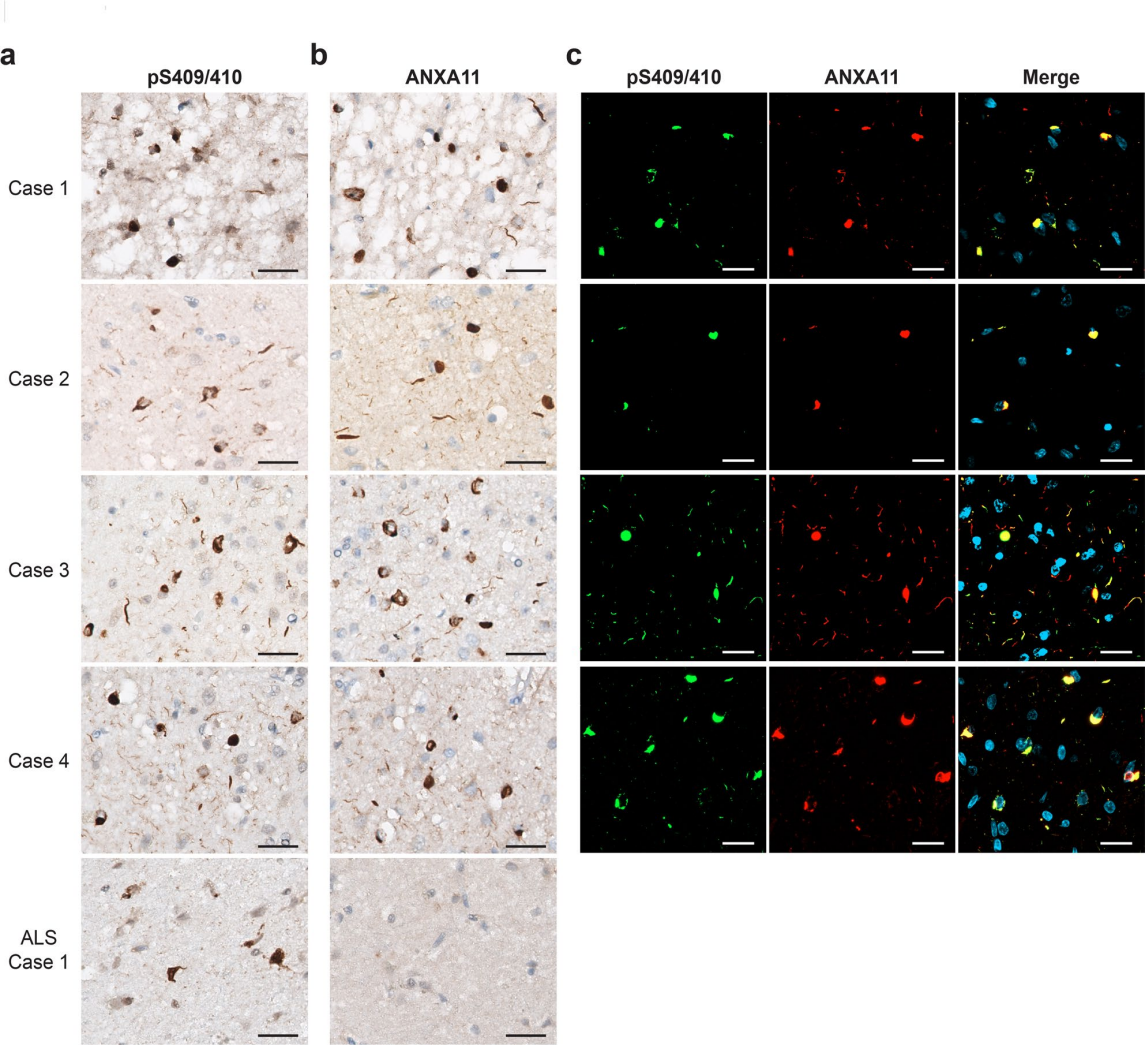
Immunohistochemistry of PLS-TDP cases with anti-pTDP-43 (pS409/410) and anti-annexin A11 (ANXA11) antibodies. **(a)** Neuronal cytoplasmic inclusions (NCIs) and short dystrophic neurites (DNs) positive for pS409/410 are observed in the frontal cortex of PLS-TDP. PS409/410-positive NCIs are observed in ALS. Scale bar: 25 μm. **(b)** ANXA11-positive NCIs and short DNs are observed in the frontal cortex of PLS-TDP, but not in ALS. Scale bar: 25 μm. **(c)** Double immunostaining of PLS-TDP with pS409/410 and ANXA11 antibodies shows pTDP-43 and ANXA11 are colocalized in NCIs and short DNs. Neuronal intranuclear inclusions were not observed. Scale bar: 25 μm

Immunohistochemistry of PLS-TDP cases showed pTDP-43-(pS409/410)-positive NCIs and relatively short DNs characteristic of FTLD-TDP Type A in the frontal cortex (Fig. 1a). No pTDP-43-positive NIIs were observed. Type A pathology in PLS-TDP cases was also ANXA11-positive (Fig. 1b), and comparable pathological features were observed in the basal ganglia and hippocampus (data not shown). In contrast, pTDP-43-positive NCIs in ALS were ANXA11-negative (Fig. 1ab). Double immunostaining confirmed that ANXA11 co-localized with pTDP-43 structures, indicating that these two proteins are both involved in Type A pathology (Fig. 1c). No mutations in *ANXA11* were detected in the four PLS-TDP cases used in this study.

### Biochemical characteristics of PLS-TDP different from those of FTLD-TDP Types A, B and C

**Fig. 2:**
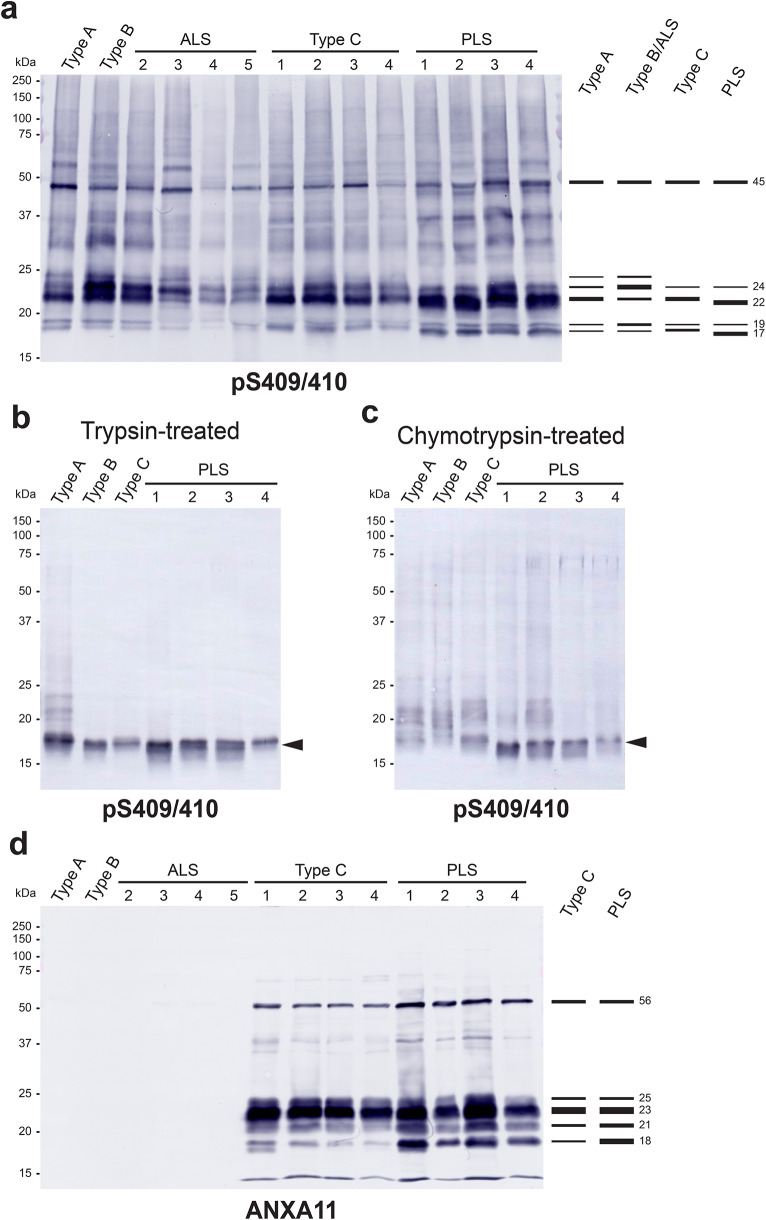
Immunoblot analysis of sarkosyl-insoluble fractions extracted from the frontal cortex of PLS-TDP cases with anti-pTDP-43 (pS409/410) and annexin A11 (ANXA11) antibodies. **(a)** Full-length pTDP-43 and C-terminal fragments (CTFs) detected in the untreated insoluble fractions are shown. The intense 22 and 17 kDa CTFs are characteristic of PLS-TDP, but are not detected in FTLD-TDP Types A, B, ALS and C. **(b)** The sarkosyl-insoluble fractions were treated with trypsin and the trypsin-resistant fragments were detected. The arrowhead indicates doublet bands at 17–18 kDa detected in PLS-TDP. **(c)** The sarkosyl-insoluble fractions were treated with chymotrypsin and the chymotrypsin-resistant fragments were detected. The arrowhead indicates the characteristic 17 kDa band of PLS-TDP, which is not detected in FTLD-TDP Types A, B, and C. **(d)** A 56 kDa band corresponding to full-length ANXA11, an intense 23 kDa band and minor N-terminal fragment (NTF) bands at 25, 21 and 18 kDa were detected in PLS-TDP and FTLD-TDP Type C. Note that the 18 kDa NTF band was more intense in PLS-TDP. ANXA11-positive bands were not detected in FTLD-TDP Types A, B and ALS

To clarify the biochemical characteristics of TDP-43 accumulated in PLS-TDP, we performed biochemical analysis of the sarkosyl-insoluble fractions extracted from the frontal cortex of four PLS-TDP cases and compared the resulting banding patterns to those of FTLD-TDP Types A, B (including ALS), and C. Immunoblotting with pS409/410 antibody showed a phosphorylated full-length TDP-43 band of 45 kDa and C-terminal fragments (CTFs) of 18 to 26 kDa that characterize each subtype as previously reported [13–15] (Fig. 2a). Interestingly, PLS-TDP cases showed 24, 22, 19, and 17 kDa CTFs distinct from those of Types A, B and C (Fig. 2a). The 22 and 17 kDa bands were most intense, while the 26, 24, 23, 19 and 18 kDa bands prominent in Types A, B and C were markedly less intense (Fig. 2a).

We also examined the banding patterns of the insoluble fractions extracted from PLS-TDP cases after treatment with trypsin and chymotrypsin. Trypsin-treated insoluble pTDP-43 showed a doublet band at around 17–18 kDa, although this was not clear in case 4 (Fig. 2b). This banding pattern is not readily distinguishable from those of FTLD-TDP Types A, B and C (Fig. 2b) [15]. In contrast, an intense 17 kDa band was detected in chymotrypsin-treated insoluble pTDP-43 (Fig. 2c). This chymotrypsin-resistant fragment detected in PLS-TDP cases was different from those in FTLD-TDP Types A, B and C (Fig. 2c) [15], suggesting that the TDP-43 filaments accumulated in PLS-TDP are structurally distinct from Types A, B and C.

In addition, immunoblotting with ANXA11 antibody detected a 56 kDa band corresponding to the full-length of ANXA11, together with N-terminal fragments (NTFs), an intense 23 kDa band and minor 25, 21 and 18 kDa bands, in PLS-TDP cases (Fig. 2d). This banding pattern resembles that detected in Type C cases [12, 18, 21], though the 18 kDa band tends to be more intense in PLS-TDP cases (Fig. 2d). Insoluble ANXA11 was not detected in Types A and B cases (Fig. 2d).

### TDP-ANXA11 heteromeric filaments in PLS-TDP cases

**Fig. 3:**
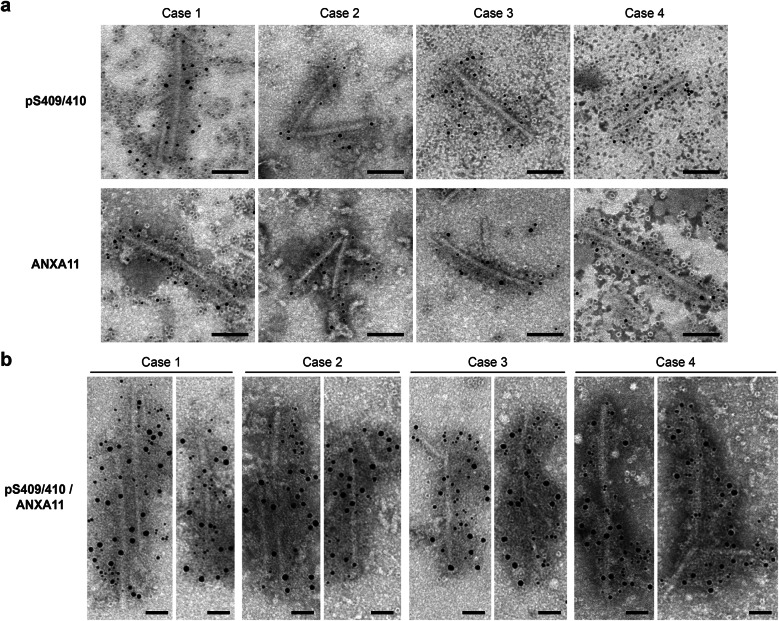
Immunoelectron microscopy of sarkosyl-insoluble fractions extracted from the frontal cortex of PLS-TDP cases. **(a)** Electron micrographs show filament structures positive for pTDP-43 (pS409/410) or annexin A11 (ANXA11), labeled with secondary antibody conjugated to 5 nm gold particles. Scale bar: 100 nm. **(b)** Double labeling of PLS-TDP filaments with pS409/410 and ANXA11 antibodies, which were labeled with 5 nm and 10 nm gold particles, respectively. Scale bar: 50 nm

To establish whether TDP-43 and ANXA11 form heteromeric filaments, we performed immuno-EM of the insoluble fraction extracted from the frontal cortex of four PLS-TDP cases. Twisted filament structures 10–15 nm in diameter were labeled with both pTDP-43 and ANXA11 antibodies (Fig. 3a), which is consistent with the characteristics of TDP-ANXA11 heteromeric filaments identified in FTLD-TDP Type C cases [12, 21]. Furthermore, double labeling with different-sized gold particles confirmed that TDP-43 and ANXA11 form heteromeric filaments in PLS-TDP cases (Fig. 3b).

## Discussion

In this study, we analyzed four cases of UMN-predominant FTLD/MND with TDP-43 pathology and found pathological and biochemical characteristics that define PLS-TDP, i.e., TDP-43- and ANXA11-positive NCIs and short DNs (Fig. 1). The banding patterns of CTFs and chymotrypsin-resistant fragments of insoluble pTDP-43 extracted from PLS-TDP cases were different from those of FTLD-TDP Types A, B, and C (Fig. 2a,c). Also, full-length ANXA11 and NTFs were detected in PLS-TDP cases by immunoblotting, as seen in Type C cases [12, 18, 21] (Fig. 2d). Furthermore, immuno-EM indicated that TDP-43 and ANXA11 form heteromeric filaments (Fig. 3). Given these results, we consider that PLS-TDP is characterized by NCIs and DNs in which TDP-43 and ANXA11 co-accumulate, with the absence of NIIs, different from Types A and B without ANXA11 pathology, and from Type C. However, if PLS is defined as a disease in which UMN selectively degenerates, there are exceptional cases with accumulation of proteins other than TDP-43 or ANXA11, as previously reported [22]. It is also possible that PLS cases presenting ANXA11-positive Type A pathology do not exhibit the clinical features of PLS, due to differences in the distribution of pathology and degenerative lesions. Further case studies are required to establish the definition and classification of PLS.

Rare familial ALS-linked genetic variants in *ANXA11* have been identified [23, 24], but their role in ALS pathogenesis has remained elusive. Robinson et al. observed co-accumulation of TDP-43 and ANXA11 not only in FTLD-TDP Type C cases but also in 3–6% of non-Type C cases, including *ANXA11* variant cases [18]. Furthermore, Arseni et al. demonstrated that TDP-43 and ANXA11 form heteromeric filaments that accumulate in Type C brains [12]. These findings strongly support the involvement of ANXA11 in FTLD-TDP pathogenesis. Our present work provides additional evidence for the involvement of ANXA11 in PLS-TDP. Our finding that PLS-TDP pathology is apparently distinct from Type B pathology, in which TDP-43 alone forms amyloid-like filaments [11], could also be critical in diagnosis and in discrimination from ALS. Although no cryo-EM study of PLS-TDP cases has yet been reported, the banding patterns of pTDP-43 and ANXA11 in PLS-TDP suggest that TDP-ANXA11 heteromeric filaments structurally different from those in Type C are present in PLS-TDP (Fig. 2). Further study of the involvement of ANXA11 in TDP-43 pathogenesis should be helpful to elucidate the mechanism of PLS-TDP onset. In addition, TDP-43 and ANXA11 in cerebrospinal fluid and plasma could potentially be valuable biomarkers for early diagnosis [25–27].

## Supplementary Information

Supplementary Material 1.

## Data Availability

All data can be obtained by contacting the corresponding authors.

## Abbreviations

PLS: Primary lateral sclerosis
MND: Motor neuron disease
UMN: Upper motor neurons
LMN: Lower motor neurons
FTLD: Frontotemporal lobar degeneration
ALS: Amyotrophic lateral sclerosis
NCI: Neuronal cytoplasmic inclusion
DN: Dystrophic neurites
ANXA11: Annexin A11
EM: Electron microscopy
