# Supplementary material for: Co-aggregation of annexin A11 and TDP-43 in FTLD/MND with primary lateral sclerosis phenotype
Source: Acta Neuropathol Commun. 2026 Jan 4;14:34. doi: 10.1186/s40478-025-02210-w (PMC12871015; doi:10.1186/s40478-025-02210-w)
Supplement: Supplementary file 1 — Supplementary Material 1. [file 40478_2025_2210_MOESM1_ESM.docx]

**Supplementary Table 1. Clinicopathological summary of FTLD-TDP cases used in this study**

PMI, postmortem interval; ALS, amyotrophic lateral sclerosis; bvFTD, behavioral variant frontotemporal dementia; FTD, frontotemporal dementia; MND, motor neuron disease; SD, semantic dementia; n.d., no data
